# Prevention and cure of murine *C. difficile* infection by a Lachnospiraceae strain

**DOI:** 10.1080/19490976.2024.2392872

**Published:** 2024-08-27

**Authors:** Juan Noriega Tejada, William A. Walters, Yanling Wang, Melissa Kordahi, Benoit Chassaing, Joseph Pickard, Gabriel Nunez, Ruth Ley, Andrew T. Gewirtz

**Affiliations:** a Center for Inflammation, Immunity and Infection, Institute for Biomedical Sciences, Georgia State University, Atlanta, GA, USA; b Department of Microbiome Science, Max Planck Institute for Biology, Tübingen, Germany; c INSERM Team “Mucosal Microbiota in Chronic Inflammatory Diseases”, CNRS UMR 8104, Université Paris Cité, Paris, France; d Institut Pasteur, Université Paris Cité, INSERM, Microbiome-Host Interaction Group, Paris, France; e Department of Pathology and Rogel Cancer Center, University of Michigan Medical School, Ann Arbor, MI, USA

**Keywords:** *Clostridioides difficile*, intestinal microbiota, fecal microbial transplant, bacteriocin

## Abstract

We sought to better understand how intestinal microbiota confer protection against *Clostridioides difficile* (*C. difficile*) infection (CDI). We utilized gnotobiotic altered Schaedler flora (ASF) mice, which lack the abnormalities of germfree (GF) mice as well as the complexity and heterogeneity of antibiotic-treated mice. Like GF mice, ASF mice were highly prone to rapid lethal CDI, without antibiotics, while very low infectious doses resulted in chronic CDI. Administering such chronic CDI mice an undefined preparation of Clostridia lowered *C. difficile* levels by several logs. Importantly, such resolution of CDI was associated with colonization of Lachnospiraceae. Fractionation of the Clostridia population to enrich for Lachnospiraceae led to the appreciation that its CDI-impeding property strongly associated with a specific Lachnospiraceae strain, namely uncultured bacteria and archaea (UBA) 3401. UBA3401 was recalcitrant to being propagated as a pure culture but could be maintained in ASF mice, wherein it comprised up to about 50% of the intestinal microbiota, which was sufficient to generate a high-quality genomic sequence of this bacterium. Sequence analysis and *ex vivo* study of UBA3401 indicated that it had the ability to secrete substance(s) that directly impeded *C. difficile* growth. Moreover, *in vivo* administration of UBA3401/ASF feces provided strong protection to *C. difficile* challenge. Thus, UBA3401 may contribute to and/or provide a means to study microbiota-mediated CDI resistance.

## Introduction

*Clostridioides difficile* (*C. difficile*) infection (CDI) remains a major public health problem, resulting in more than 30,000 excess deaths and 5 billion USD in health costs annually.^1^ The predominant, arguably essential, risk factor for CDI is the recent consumption of antibiotics, which decimate the resident gut microbiota that normally provide profound colonization resistance against *C. difficile*. The ability of a healthy microbiota to impede *C. difficile* colonization is also evidenced by the efficacy of fecal microbiota transplant (FMT) in preventing CDI recurrence.^2^ Its efficacy notwithstanding, FMT has significant pitfalls, particularly those stemming from the engraftment of an undefined donor microbiome.^3^ Indeed, a variety of microbiota members have been implicated with medical conditions ranging from metabolic syndrome and inflammation to cancer and atherosclerosis, highlighting that FMT can transfer microbes deleterious to a patient’s health.^4^ Although screening for known pathogens can reduce the risk, the incomplete information on how microbes may affect their host’s health makes it difficult to prevent the engraftment of deleterious microbes. As the death of two immunocompromised patients and the hospitalization of others due to enteropathogenic *E. coli* that evaded pathogen screening shows, despite precautions, FMT poses a significant degree of risk.^5^ Thus, there is great interest in developing a narrowly targeted, fully defined microbe(s) that would allow for safer prevention of CDI recurrence.

Several studies have identified microbiota residents that associate with and are capable of mediating colonization resistance to CDI in mice.^5,6^ Such findings notwithstanding, specific bacteria that contribute to such colonization resistance, and discern how they do so, remain poorly defined. One factor that has stymied the study of microbiota−*C. difficile* interactions is the extensive degree of basal heterogeneity in microbiota composition among different colonies of mice.^7,8^ This heterogeneity is exacerbated by the rapid transient variability in the microbiota induced by antibiotics, which are typically used in abundance to make mice prone to *C. difficile* colonization.^9^ Microbiota heterogeneity capable of modulating proneness to *C. difficile* can occur in a variety of taxa but one characterized example of this notion is our observation that mice from a variety of sources variably harbor murine *C. difficile*, which is maintained at very low levels so as not to impact the basal phenotype but can bloom upon antibiotic treatment and alter the response to experimentally administered *C. difficile* isolates.^10^ Microbiota heterogeneity and complexity have also stymied understanding of the mechanism by which select taxa impede *C. difficile* colonization. Such lack of mechanistic understanding has precluded the rational engineering of bacteria to optimally impede *C. difficile*. More generally, the inability to understand mechanisms whereby limited numbers of taxa impede *C. difficile* has made it difficult to understand how complex microbiotas normally provide resistance to CDI.

Germfree (GF) mice are one possible platform to study how specific bacteria interact with *C. difficile in vivo*, but a detrimental aspect of this approach is that GF mice have a range of abnormalities in metabolism and immune system development. Hence, we sought to surmount this hurdle via the use of gnotobiotic mice harboring a group of bacteria known as altered Schaedler flora (ASF).^11^ ASF mice have a defined minimal, 8-member microbiota composed of *Parabacteroides goldsteinii*, *Eubacterium plexicaudatum*, *Schaedlerella arabinosiphila*, *Pseudoflavonifractor* sp., *Clostridium* sp., *Mucispirillum schaedleri*, *Ligilactobacillus intestinalis*, and *Lactobacillus murinus*.^11^ This microbial consortium restores relatively normal gut physiology and immune function such that ASF mice lack many of the metabolic and immunologic abnormalities exhibited by GF mice.^11^ We herein report that, like GF mice, ASF mice were highly prone to *C. difficile* colonization, without the need for antibiotics. We further report that ASF mice were protected by Lachnospiraceae species, particularly uncultured bacteria and archaea (UBA) 3401 (classified as COE1 sp002358575 in the Genome Taxonomy Database).

## Methods

### Mice

*C57BL/6j* mice were maintained in conventional specific pathogen-free housing or gnotobiotic isolators, GF or ASF), as previously described.^12^ All mice were maintained with an autoclaved 5010 rodent diet and autoclaved water. Experiments utilized 6–12-week-old male and female mice, which were caged by sex.

### Preparation and titration of C. difficile

*Clostridioides difficile*, strain VPI10463, was cultured in brain heart infusion (BHIS) broth (Millipore cat. no. 53286) in a mix of 90% nitrogen, 5% hydrogen, and 5% CO_2_ atmosphere as previously described.^13^ Quantification of spore solution CFU is carried out via serial dilution on taurocholate–cefoxitin–cycloserine–fructose agar (TCCFA) plates chosen for their ability to exclude microbes other than *C. difficile* and germinate *C. difficile* spores to ensure only viable VPI10463 spores were counted.^13^ Spores were diluted serially to generate the desired infectious dose. The lack of a broth form of TCCFA and the potential interference of antibiotics with growth assay results resulted in the use of BHIS media.

### Acute C. difficile challenge

Gnotobiotic mice, either ASF or GF, were administered an experimental treatment or vehicle and challenged after 72 hours. Mouse condition was monitored throughout the experiment, and shedding, weight, inflammatory cytokines, and fecal VPI shedding were monitored for 2 weeks. Autoclaved cages and food were used throughout the experiment.

### Chronic CDI ASF mouse model

6–8-week-old ASF mice were moved from gnotobiotic isolators to sterilized cages and maintained with autoclaved food/water. They were administered 10^2^ spores/mouse. Mice administered 10^2^ *C. difficile* spores via oral gavage were monitored and provided with oral rehydration solution to reduce mortality. VPI levels stabilized 2 weeks after infection allowing for a window of opportunity to test therapeutic interventions under controlled conditions. Mice were monitored for recovery as described for the acute challenge model.

### DNA isolation

Fecal samples were diluted with PBS + 0.1% Tween 20 (100 mg/ml), and 250 μl of the homogenate was transferred for extraction. Lysis was performed via bead beating and heating at 64°C for 10 minutes while incubated with 10% sodium dodecyl sulfate, 300 μl of Tris–EDTA saturated phenol (Thermo Fisher cat. no. 327125000), and 200 μl of Tris buffer (Millipore cat. no. 648314). Samples were subsequently centrifuged for 5 minutes at 12,000 *g* at 4°C, and the aqueous supernatant was collected. Phenol–chloroform extraction was executed via the addition of 450 μl of phenol–Tris followed by centrifugation for 5 minutes at 12,000 *g* at 4°C and subsequent collection of the aqueous supernatant and incubation with 700 μl of chloroform (Sigma Aldrich cat. no. 34854) and centrifugation. A 250 μl aqueous layer was collected and dissolved in 1000 μl of pure ethanol (Decon cat. no. 2716) and 30 μl of sodium acetate (Thermo Fisher. cat. no. AM9740) and stored at −20°C for 30 minutes. A DNA pellet was subsequently yielded by centrifugation at 12,000 *g* at 4°C for 30 minutes; pellets were subsequently rinsed with 75% ethanol. DNA purity was tested following resuspension with 100 μl of water. If purity assessed by 260:280 was unacceptably low, an additional purification via precipitation with ethanol and sodium acetate was performed.

### Quantification of VPI10463

Detection and quantification of VPI10463 were carried out via the use of 2.5 μl of sample DNA of acceptable quality under the qPCR methods previously described.^10^

### Assessment of symptom severity

Mice were placed individually in small chambers, and an experienced mouse researcher, blinded to the study criteria, was asked to examine and score each mouse for the following symptoms with the following scoring criteria: dehydration (1 if skin was bunchy/loose else 0), grimace (1 if an abnormal facial expression was present else 0), rectal inflammation (1 if overt 0.5 if subtle, else 0), and mobility (0, 0.5, 1, 1.5, or 2 for normal to severe lack of mobility). Symptom scores were added to produce a symptom severity score for each mouse.

### Generation of UBA3401-specific primers

Partial UBA 3401 sequence in NCBI (NCBI: txid1952033) was compared in bioinformatics via Geneious software (NZ) with other members of the Lachnospiraceae and Clostridia taxa. Regions of the UBA3401 genome were analyzed to find sequences not conserved with other bacteria with a UBA3401 sequence homologous to leucyl/phenylalanyl-tRNA-protein transferase, dissimilar in sequence to all other bacteria assessed. UBA3401-specific primers were designed, 2241F (5’-ATGGGAGCCCTGTTGTCCTA-3’) and 2669 R (5’-AATCGCCGTTTTACCAACGC-3’), with a predicted 429BP amplicon. Testing sequencing-confirmed UBA3401-positive samples indicated a 429BP product, indicating the presence of UBA3401.

### Detection of UBA3401

2.5 μl of purified sample DNA was utilized for qPCR with 12.5 μl of Qiagen Quantinova SYBR Green master mix (cat. no. 208052) per reaction, 8 μl of mq water with 1 μl of 2241F and 1 μl of 2669 R primers, respectively. Amplification was carried out under an initial 10-minute 95°C denaturation followed by 60 cycles of 15 seconds of 95C denaturation and 60 seconds of 60°C annealing-extension; followed by a final 60-second 95°C and 60-second 60°C step. A melt curve was performed under 60 iterations of a 10-second 55°C and 0.5-second 95°C step. A melt curve peak in the range of 80–81°C is suggestive of UBA3401 presence. Confirmation of UBA3401 presence was achieved via gel electrophoresis with a 429BP product corresponding to a unique UBA3401 sequence being highly specific to UBA3401. Ct threshold in UBA3401 positive cycles is inversely correlated with the abundance of UBA3401.

### Bulk culture of cecal Lachnospiraceae

0.2 μm of filtered 0.2% cystine PBS along with autoclaved Lachnospiraceae-selective BHIS agar plates (Millipore cat. no. 70138 and Y1625) enriched with 2 ml/l gentamicin (Sigma Aldrich cat. no. G1264) and 1 ml/l aztreonam (Sigma Aldrich cat. no. PHR1785-500 MG) were reduced overnight in an AS580 anaerobic chamber. An ASF mouse was obtained, which had received the simplified consortium 28 days (d) prior and was housed inside an Isocage and euthanized under aseptic conditions. Cecal contents were suspended in 100 mg/ml reduced PBS–cystine in an anaerobic chamber. An additional 1:100 dilution was performed with the cecal homogenate with the diluted solution plated on the Lachnospiraceae selective plates and incubated at 36°C for 7 d. Resulting colonies were harvested in bulk and stored in anaerobic aseptic 30% glycerol PBS with the resulting solution designated the Lachnospiraceae consortium.

### Enrichment of UBA3401-relative abundance

Fecal samples were obtained from ASF mice that had received UBA3401. Those samples were heated to 55°C, exposed to chloroform, and subsequently administered to a gnotobiotic mouse. 14 d post-administration, fecal samples were collected from the mouse, and the presence of UBA3401 was confirmed. Subsequently, the mouse was administered kanamycin (Sigma Aldrich cat. no. PHR1487) at 50 mg/L via drinking water followed by withdrawal of the drug after 48 hours. 72 hours were allowed to elapse before fecal samples were tested to confirm that the abundance of UBA3401 – predicted to be inherently resistant to aminoglycosides like most anaerobes^14^ – was not depleted. Samples were subsequently collected and used for additional iterations to reduce the number of non-UBA3401 taxa.

### Cultivation of UBA3401 consortium and propagation in ASF host

Cecal contents of UBA3401-positive ASF mice were suspended in anaerobic 0.2% cystine PBS. The suspension was serially diluted and streaked onto BHIS plates supplemented with 1 mg/l gentamicin and 10 ml/l taurocholate 10% inside an AS580 anaerobic chamber. Plates were allowed to grow under anaerobic conditions at 36°C for 10 d after which individual colonies were harvested and homogenized in 30% glycerol PBS for storage at −80°C. Stock from the colonies was screened for the presence of UBA3401. Two samples – a trio of overlapping colonies and a bulk collection of colonies from the same plate – tested positive. Both samples were administered to ASF mice held inside Isocages. Fecal samples were collected at 1-week intervals and screened to confirm successful UBA3401 engraftment.

### Colonization of ASF mice via cecal administration

An ASF mouse donor shown by metagenomic sequencing to be predominant in UBA3401 and shown by qPCR to possess a very high absolute quantity of UBA3401 was euthanized. The donor’s cecal materials were immediately collected and resuspended in anaerobic 30% glycerol, 0.1% cystine PBS at a 100 mg/ml concentration. Fecal samples were obtained at 3 d intervals to track the engraftment and expansion of UBA3401. The presence of UBA3401 was confirmed by PCR, and its growth was tracked until an acute challenge with 10^5^ VPI10463 spores at 19 d post-UBA3401 administration.

### UBA feces extraction and in vitro VPI10463 growth assay

A sample of 2 g of feces from UBA3401 consortium mice was homogenized in 600 μl PBS via 1-minute bead beating. 1 ml of chloroform was added to the sample, and a subsequent 5-minute bead beating step was used to promote diffusion of hydrophobic compounds into the chloroform layer. Both aqueous and chloroform layers were subsequently passed through a 100 μm cell strainer, aliquoted into three tubes for each type of extraction, and subjected to vacufuge (Eppendorf Concentrator) treatment at 30°C for 4 hours under high vapor pressure mode. Chloroform extracts were checked to ensure chloroform had completely evaporated leaving a solid precipitate, which was dissolved in 1 ml PBS via 1-minute bead beating. Samples were passed through a 0.22 μm syringe filter. Identical methods were utilized to create aqueous and chloroform extract ASF control samples.

Aliquoted extracts were combined with 1.5 ml BHIS broth to create replicate groups of aqueous and chloroform UBA or ASF extracts. Suppression of VPI10463 was assayed by inoculating each extract-BHIS sample with 10 μl of broth containing early log phase VPI prepared 24 hours prior by inoculation with 10 μl log phase VPI10463 broth culture. All steps were carried out in an anaerobic chamber under standard anaerobic conditions. Immediately following inoculation of extract-BHIS tubes, 250 μl of sample was collected and DNA was extracted. Additional collections were carried out at 24-hour intervals, and VPI10463 was quantified via qPCR.

### 16S sequencing

DNA was extracted and purified from the frozen stool and sputum samples using the DNeasy 96 PowerSoil Pro QIAcube HT kit, supplemented with PowerBead Pro Plates (Qiagen). The V3–V4 region of 16S rRNA genes was amplified using the following primers: 341F 5′TCGTCGGCAGCGTCAGATGTGTATAAGAGACAGCCTACGGGNGGCWGCAG-3′; 805 R 5′GTCTCGTGGGCTCGGAGATGTGTATAAGAGACAGGACTACHVGGGTATCTAATCC-3′. PCR products of each sample were purified using Ampure XP magnetic purification beads. An index PCR was performed to attach dual barcodes and Illumina sequencing adapters using the Nextera XT Index kit (Illumina). Final PCR products were verified on a 1.5% DNA agarose gel, purified again using Ampure XP magnetic purification beads, and quantified using Pico dsDNA assay (Invitrogen). An equal molar amount of each sample was then combined as the library. The library was diluted and spiked with 5% PhiX control (Illumina), sequenced by the Illumina MiSeq Sequencing System (2 × 250 bp), and demultiplexed via the Illumina software. Due to quality issues with read 1, only read 2 was used for the 16S amplicon analysis.

### Metagenomic sequencing

Samples, apart from the hybrid assembly samples, were processed (sample preparation and bioinformatic processing) as described previously.^6^ Briefly, 1 ng of DNA, extracted with the boiling lysis approach described above, was used for Nextera Tn5 tagmentation, followed by a 10-cycle PCR reaction to add barcodes and cleanup with Mag-Bind TotalPure NGS beads (Omega Biotech, Norcross, USA). Fragments of 400–700 bp were selected with BluePippin, followed by sequencing on an Illumina HiSeq3000 instrument with 2 × 150 paired-end sequencing. Two samples, an ASF and ASF + UBA3401 consortium from mouse ceca, were used for hybrid assembly (Illumina short read and PacBio reads). These samples were collected from mice and stored at room temperature for 7 d with the Norgen Biotek Corporation Stool Nucleic Acid Collection and Preservation Tubes (cat. no. 45660) during transport, and then at −20°C until processing. DNA was extracted using the ZymoBIOMICS DNA MiniPrep Kit (cat. no. D4300) with a 10-minute bead-beating step to minimize DNA shearing. DNA from this was used for both short-read sequencing as above but with an Illumina NextSeq sequencing system, and long reads were sequenced from these samples with the Sequel II (Pacific Biosciences).

Trimming and QC of Illumina short reads, taxonomic assignments, and metagenome assemblies were carried out as described by Youngblut et al.^6^ except as noted in the following text for software versions, parameters, and reference databases. Taxonomic assignments and estimated abundances were calculated with Kraken2 and Bracken 2.2, using the Genome Taxonomy Database version 202^15^ as the reference database. Functional profiling was carried out on the same reads using HUMAnN 3.0 v3.0.0.alpha.3 A Struo2.^16–19^ Reference database v207 was used for UniRef50 classification of reads. Default settings for HUMAnN were used, except for the following: bowtie2 options = –very-sensitive, diamond options = –mid-sensitive – query-cover 80 –id 50 –max-target-seqs 1 –block-size 4 –index-chunks 2, evalue threshold = 0.001, and prescreen threshold = 0.01. PacBio data were demultiplexed and processed using the Lima software (version 2.7.1) with default settings. Demultiplexed PacBio and NextSeq short read data were used as input for Spades 3.15.4,^20^ using the –PacBio and –k 21,33,55,77 parameters to generate hybrid assemblies. Antimicrobials were detected with AntiSMASH version 6.0.1.^21^ Kruskal–Wallis and multiple comparison tests were performed using the kruskal.test and p.adjust (method=”holm”) R functions.

## Results

### ASF mice are prone to acute and chronic C. difficile infection

**Figure 1.**
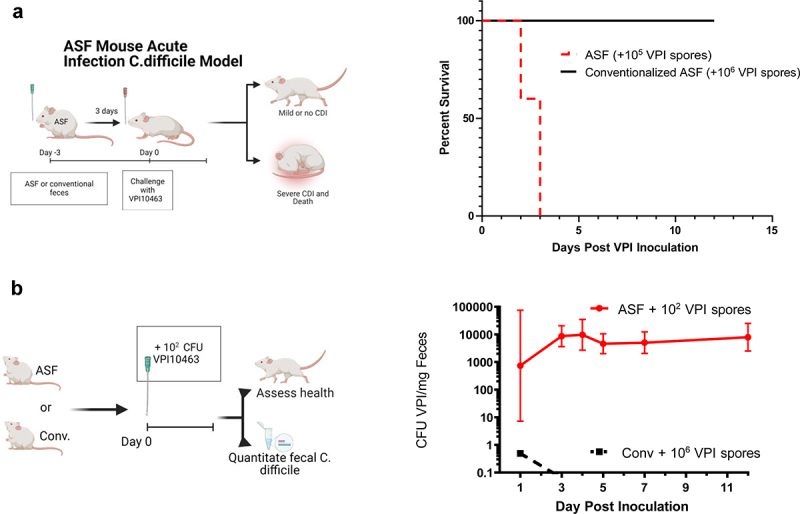
Altered Schaedler flora (ASF) mice were prone to acute and chronic CDI.

We investigated the potential of ASF mice, which lack the immune and metabolic defects of GF mice,^11^ to serve as an antibiotic-free model of *C. difficile* infection (CDI). Specifically, we assessed proneness to CDI in ASF mice vs. ASF mice that had been “conventionalized” via administering them a fecal suspension generated from conventional mice. We observed that ASF mice were indeed exquisitely sensitive to *C. difficile* exposure uniformly exhibiting mortality following inoculation with 10^5^ spores of *C. difficile* VPI10463, a commonly used dose for antibiotic-treated mice (Figure 1(a)), while conventionalized ASF mice exhibited colonization resistance to CDI similar to what would be expected for truly conventional mice. Specifically, administering conventionalized ASF mice a 10-fold-higher dose (10^6^ spores) of *C. difficile* VPI10463 did not result in colonization (fecal *C. difficile* was undetectable by PCR 2–4 d post-inoculation), symptomatic disease, or mortality (Figure 1(a)), thus indicating that strong colonization resistance had been provided by the transplanted conventional microbiota. ASF mice mostly survived (75%) a lower dose *C. difficile* challenge, namely 100 spores per mouse, but remained chronically infected as indicated by levels of *C. difficile* genomes in their feces as measured by a PCR assay standardized via comparison to samples of known *C. difficile* CFU (Figure 1(b)). In contrast, and in accord with numerous published studies, truly conventional mice administered a much higher dose of *C. difficile*, namely 10^6^ spores, only barely and transiently displayed detectable levels of fecal *C. difficile*. The ASF mice chronically infected with *C. difficile* exhibited an overtly ill appearance characterized by hunched posture and low activity (both of these symptoms were exhibited by all 9 ASF mice and 0 of 5 conventional mice in Figure 1(b)) although histopathologic analysis did not indicate overt gut inflammation (a pathologist examined tissue from 5 ASF mice with chronic symptomatic CDI but did not identify any features of overt inflammation reminiscent of CDI). We investigated whether chronic *C. difficile* infection could also be attained in GF mice. We subjected 11 GF mice to low-dose *C. difficile* challenge. 9 of the mice died within 3 d inoculation. The remaining mice exhibited a severely ill appearance over the course of the 10 d we monitored them. Thus, we reasoned that ASF mice were not only a more physiologic model but also a more practical model to study how specific bacterial taxa might impede *C. difficile in vivo*.

(a) Acute CDI model. Experimental schematic and Kaplan–Meier survival plot following acute CDI. ASF mice were administered feces from ASF or conventional mice and, 3 d later, inoculated with 10^5^ or 10^6^ *C. difficile* VPI10463 spores, respectively (*n* = 10). (b) Chronic *C. difficile* model. Experimental schematic and *C. difficile* levels. Conventional or ASF mice were inoculated with 10^6^ or 10^2^ VPI10463 spores. Health and fecal *C. difficile* levels were monitored. Data are geometric means ± 95% CI, from a single experiment (*n* = 9 for the ASF group and 5 for the conventional mice group).

### Clostridia species provide ASF mice colonization resistance to C. difficile

**Figure 2.**
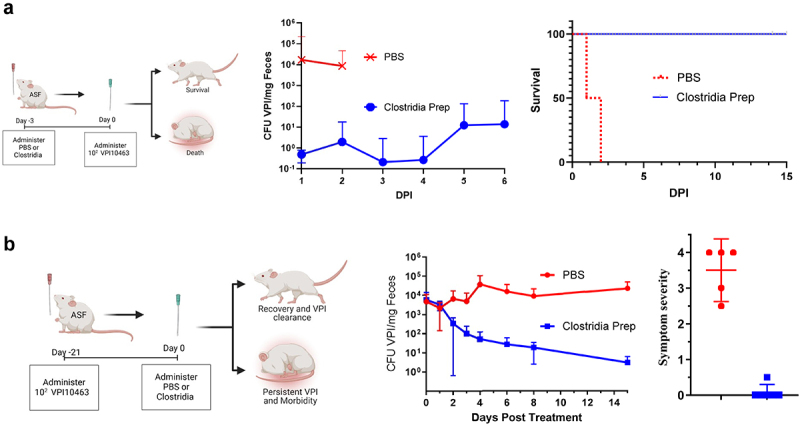
Clostridia consortium protected ASF mice against acute and chronic CDI.

We next sought to investigate the potential ability of select classes of commensal bacteria to impact acute and/or chronic *C. difficile* infection in ASF mice. Specifically, we examined the impact of a previously described crude preparation of Clostridia, which had been isolated from conventional mice.^22^ This Clostridia preparation contains hundreds of species from several families including in order of relative abundance: Lachnospiraceae (32%), Ruminococcaceae (29%), Bacillaceae (7%), Enterobacteriaceae (4%), Enterococcaceae (3%), Eubacteriaceae (3%), and unclassified (13%). Administration of the Clostridia preparation confers colonization resistance to gram-negative facultative aerobic pathogens *S. typhimurium* and *C. rodentium* but which taxa mediated the protection was not determined.^22^ We herein observed that this Clostridia preparation also protected ASF mice against acute *C. difficile* challenge. Specifically, administration of the Clostridia preparation to ASF mice 7 d prior to inoculation with 10^5^ *C. difficile* spores reduced fecal levels of *C. difficile* by over 4-logs and conferred 100% survival through 12 d, at which time we stopped specifically monitoring it, whereas all untreated ASF mice died within 3 d of *C. difficile* administration precluding sample collection past the second day (Figure 2(a)). We next administered the Clostridia prep to ASF mice with established chronic *C. difficile* infection, achieved via a low-dose inoculation 21 d prior. Such chronically infected mice that received the Clostridia preparation displayed a stark drop in *C. difficile* levels within a week and thereafter displayed only low levels of this pathogen (fewer than 100 *C. difficile* genomes per mg of feces) (Figure 2(b)). In contrast, mice administered vehicle (PBS) continued to display high levels of *C. difficile* in their feces. Furthermore, visual assessment of the health of these mice (described in Methods) revealed that the reduction in *C. difficile* levels following administration of the Clostridia preparation was associated with restoration of indices of health readily evident by a near-complete elimination of visible disease symptoms. These results indicated that the use of ASF mice to model acute and chronic *C. difficile* infection could serve to investigate microbiota-mediated amelioration of this condition.

(a) ASF mice were administered PBS or the Clostridia preparation 3 d prior to challenge with 10^5^ VPI10463 spores. Levels of fecal VPI and survival were monitored on indicated days post-inoculation. Data are means ± S.E.M. (*n* = 4, 2 M/2 F for PBS group and 5 2 M/3 F for Clostridia group). (b) ASF mice were inoculated with 10^2^ VPI10463 spores to establish a chronic infection. 21 d later, mice were administered PBS or Clostridia prep by oral gavage (referred to as d0 post-treatment). Fecal *C. difficile* levels were monitored on indicated day post-treatment. 14 d later, mice were given a symptom severity score as described in methods. Data are means ± S.E.M. (*n* = 5, 2 M/3 F for PBS and 6, 3 M/3 F, for Clostridia prep).

### Protection against C. difficile associated with Lachnospiraceae

**Figure 3.**
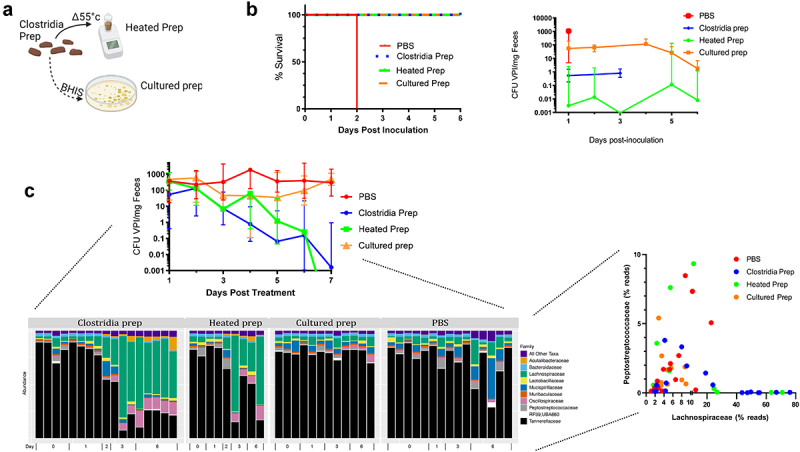
Clostridia prep’s protection against CDI is independent of host immunity and is associated with Lachnospiraceae.

We presumed that the protection against CDI by the Clostridia preparation likely reflected that the microbes it contained, and/or the products it produced, were acting directly upon *C. difficile*. Yet it also seemed conceivable that the Clostridia preparation might have reduced *C. difficile* levels via activation of the immune system. Hence, to investigate whether the adaptive arm of the immune system played a role, we used GF Rag1KO mice, which are broadly deficient in adaptive immunity. As expected, all mice in the control (PBS) group succumbed to *C. difficile* by 48 hours post-inoculation. In contrast, all GF Rag1KO mice that received the Clostridia preparation survived (Figure 3(b)) thus arguing against a role for adaptive immunity in mediating the Clostridia preparation’s protection against CDI. We next turned our attention to discerning the key components of the Clostridia preparation that mediated its ability to protect against *C. difficile*. The Clostridia preparation is a chloroform generated derivative of conventional mouse feces and is thought to be comprised of hundreds of microbial species with members of the class Clostridia predominating.^6^ The Clostridia preparation itself is maintained by administering it to GF mice and then isolating their feces. In an attempt to capture the protective microbes in cell culture, fecal pellets of GF mice that had been administered the Clostridia preparation were anaerobically incubated in BHIS for 3 d and then plated on BHIS agar. All colonies were collected, pooled, and replated. The collection of pooled colonies on the second plate was referred to as the cultured Clostridia prep (Figure 3(a)). This cultured-Clostridia prep also protected GF Rag1KO mice against *C. difficile*-induced mortality although the extent to which it lowered fecal *C. difficile* levels were much less than what were observed for the starting Clostridia preparation (Figure 3(b)). We also generated a heated version of the Clostridia preparation by incubating a fecal suspension of it at 55°C for 30 minutes prior to initially plating it on BHIS agar. This heated Clostridia prep protected mice against acute CDI at least as well as the original Clostridia preparation as assessed by both survival and fecal *C. difficile* levels. Collectively, these results suggested that the Clostridia preparation’s ability to protect mice against *C. difficile* was mediated by microbes that had limited capacity to grow under standard anaerobic culture conditions but were capable of generating heat-stable spores that could germinate in the mouse intestine.

(a) Schematic of how cultured clostridia prep and heated clostridia prep were generated. (b) GF Rag1KO mice were administered the Clostridia prep or fractionated version thereof as indicated. 7 d later, mice were inoculated with 10^3^ VPI10463 spores. Survival and fecal *C. difficile* levels were monitored. Data are means ± S.E.M. (*n* = 4). Some groups of mice failed to produce fecal samples on some individual days. (c) ASF mice with established chronic CDI (as a result of inoculation with 10^2^ VPI10463 spores of 21 d prior) were administered the Clostridia preparation, or derivative thereof, as indicated. Fecal samples were subjected to qPCR to measure *C. difficile* levels. Data are means ± S.E.M. (*n* = 3–5). Select samples were also subjected to shotgun metagenomic sequencing. The bar chart shows taxonomy at the family level at the indicated time point following administration of the indicated treatment. The point plot shows the percentage of reads assigned to Lachnospiraceae or Peptostreptococcaceae (the family to which *C. difficile* belongs) for individual samples from each condition.

We next administered the original Clostridia preparation, the heated Clostridia prep and the cultured Clostridia prep to ASF mice with established chronic *C. difficile* infections. Analogous to results in the acute model, the cultured Clostridia prep had modest impacts on *C. difficile* levels, while the original Clostridia preparation and its heated derivative both lowered *C. difficile* levels within 7 d of their administration (Figure 3(c)). We next analyzed the microbiotas of these mice via shotgun metagenomic sequencing over time, seeking to identify taxa whose appearance was associated with the disappearance of *C. difficile*. Species-level analysis of the sequencing data did not yield consistent trends. Hence, we focused on family-level analysis, which revealed that, not surprisingly, Tannerellaceae, the family to which most ASF bacteria, including the predominant ASF microbe, *Parabacteroides goldsteinii* (ASF519), belong was prominent in all samples. As further expected, Peptostreptococcaceae, the family to which *C. difficile* belongs, was also apparent in these chronically infected mice. More interestingly, we observed that the appearance of Lachnospiraceae was associated with the disappearance of Peptostreptococcaceae as the treated mice resolved CDI. More specifically, Lachnospiraceae was a predominant family (43–76% of reads) in mice receiving that had cleared *C. difficile* but was a relatively minor component of the microbiota (under 22%) in the mice that had not. This inverse correlation between *C. difficile* and Lachnospiraceae was appreciable by plotting the relative amounts of Lachnospiraceae and *C. difficile* in individual samples. This led us to hypothesize that Lachnospiraceae species might provide colonization resistance against *C. difficile*. To gain potential insight into how Lachnospiraceae might impede *C. difficile*, we performed functional profiling of the shotgun sequencing data via HUMAnN3 to generate UniRef50 clusters (Figure S1). This approach did not show the clear contrast exhibited by the taxonomic profile but did reveal a shift over time in which the expulsion of *C. difficile* was associated with more unmapped reads on day 6 (mean ± SD unmapped reads were 37.5 ± 3.90 vs. 34.0 ± 10.5% for mice receiving Clostridia preparation and heated Clostridia prep vs. cultured Clostridia prep and PBS groups, respectively), although this difference, and all other UniRef50 clusters, were not statistically significant when tested via the Kruskal–Wallis test and Holm multiple comparison correction.

### Lachnospiraceae-mediated protection against C. difficile is associated with UBA3401

**Figure 4.**
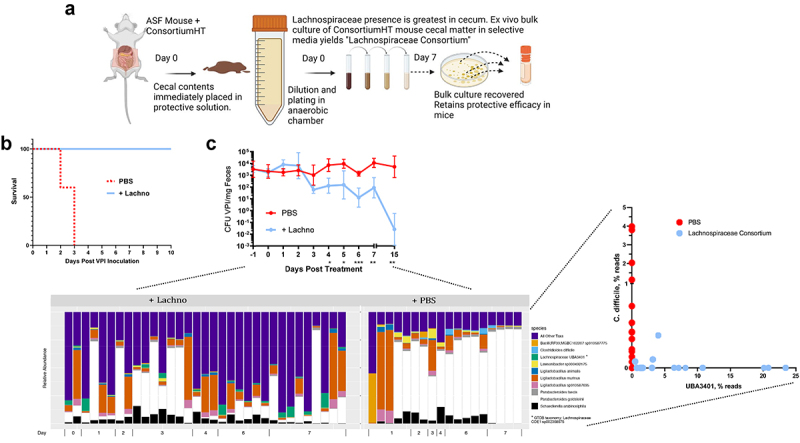
Protection against CDI by a Lachnospiraceae-enriched consortium associates with levels of Lachnospiraceae UBA3401.

We reasoned that, although our ability to propagate the *C. difficile* suppressing microbes *ex vivo* was quite limited, it nonetheless might provide an opportunity to investigate our hypothesis that Lachnospiraceae had suppressed *C. difficile* in our model. We isolated cecal contents from ASF mice that had been administered the heat-treated singly passaged Clostridia prep and sought to culture it using conditions known to favor Lachnospiraceae. Accordingly, testing of an array of individually selected colonies by 16S sequencing confirmed the predominance of Lachnospiraceae family members. We then pooled the colonies collected from individual plates, generating glycerol stocks that were stored at −20°C, and hereafter referred to as a Lachnospiraceae-enriched consortia (Figure 4(a)). Analogous to the original Clostridia preparation, the Lachnospiraceae-enriched consortia protected ASF mice against high-dose *C. difficile* challenge (Figure 4(b)) and, moreover, reduced *C. difficile* levels in ASF mice with previously established chronic *C. difficile* infections (Figure 4(c)). Functional profiling of these data (Figure S2(a)) reiterated the previously observed trend of increased unmapped reads in the UniRef50 clusters on days 6 and 7 in mice that expelled *C. difficile* (% mean ± SD unmapped reads were 33.9 ± 5.81 vs. 21.8 ± 0.869% for Lachnospiraceae and PBS groups, respectively). One significant UniRef50 cluster was found after Holm multiple comparison correction, E2ELK6, a putative *Clostridium* phage phiCD6356 protein, which was undetected in all mice receiving the Lachnospiraceae consortium and non-zero in all PBS control mice (Figure S2(b)). The association of this UniRef50 cluster likely reflects the promulgation of its host *Clostridium* spp. in the unprotected communities.

(a) Schematic illustration of approach used to generate a Lachnospiraceae-enriched consortium from the Clostridia prep. (b) ASF mice were administered PBS or a Lachnospiraceae-enriched consortium. 7 d later, they were challenged with 10^5^ VPI10463 spores (*n* = 5). (c) ASF mice with established chronic CDI (as a result of inoculation with 10^2^ VPI10463 spores 21 d prior) were administered PBS or a Lachnospiraceae-enriched consortium. Fecal samples were subjected to qPCR to measure *C. difficile* levels. Data are means ± S.E.M. (*n* = 6, 3 M/3 F). Select samples were also subjected to shotgun metagenomic sequencing. The bar chart shows species level taxonomy at the indicated time point following the administration of indicated treatment. The point plot shows the percentage of reads assigned to *C. difficile* and UBA3401 for individual samples from each condition.

To identify specific Lachnospiraceae species that had potentially helped clear *C. difficile*, feces from these mice were subjected to shotgun metagenomic sequencing, hoping the reduced complexity relative to the previous samples might aid species level analysis. Indeed, species-level taxonomy bar charts revealed the reduction in *C. difficile* levels in mice that had received the Lachnospiraceae-enriched consortia was associated with the appearance of a Lachnospiraceae species, namely UBA3401 (ANI is ~ 99.4%, as calculated by FastANI is ~ 99.4%), a bacterium, which, as its name implies, is only known to exist as one of the thousands of intestinal bacterial genomes assembled from existing databases of shotgun sequencing.^23^ The inverse association of UBA3401 and *C. difficile* was most readily appreciated via a point plot of the relative abundance (% reads) of these species in individual samples. Hence, we hypothesized that UBA3401 might have the capacity to impede *C. difficile*.

### Generation of ASF/UBA3401-colonized mice

**Figure 5.**
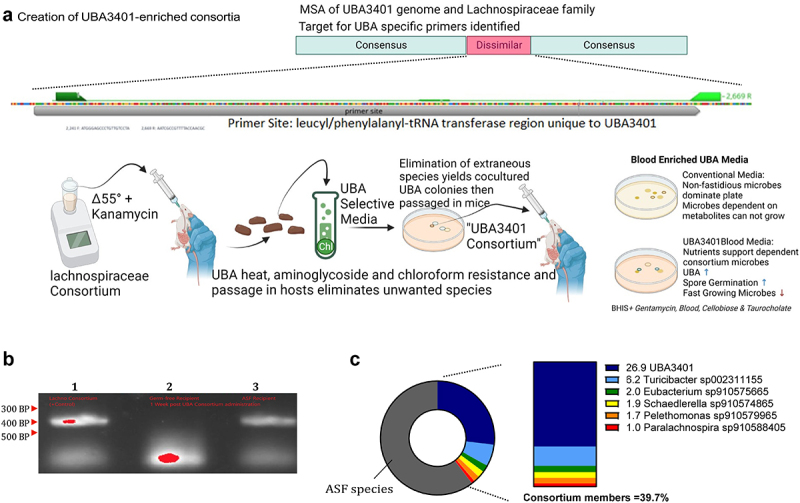
Creation of UBA3401-enriched consortium in ASF mice.

The previously reported partial UBA3401 genome (NCBI: txid1952033) was used to design primers capable of specifically recognizing the presence of this bacterium. We identified a region of the publicly available UBA3401 genome (GCA_002358575.1), namely bases 2241–2669, that was distinct from other microbes including those of the Lachnospiraceae family (Figure 5(a)). Primers targeting this region successfully amplified the expected 429 BP fragment from fecal samples of ASF mice that had received the Lachnospiraceae-enriched consortium (Figure 5(b)). Such a PCR product was not observed when using feces from conventional or conventionalized mice although quantitative (q) PCR found amplification at high cycle numbers, suggesting UBA3401 is present at low levels in non-manipulated microbiomes and that our gel-based assay has a higher threshold of UBA3401 detection. As expected, those samples showing the presumed UBA3401-specific PCR product on gels displayed qPCR positivity at relatively low Ct numbers, which, when compared to Ct values obtained with universal 16S primers, served to identify the samples with the greatest relative abundance of UBA3401. We next used these PCR assays to monitor the results of an array of attempts to cultivate UBA3401*in vitro*. Feces and cecal contents, suspended in deoxygenated PBS, were cultured, anaerobically, under a wide variety of conditions, and numerous colonies screened for presence of UBA3401. In contrast to most conditions, which failed to yield any positive colonies, culture on BHIS enriched with defibrinated sheep blood, taurocholate, and cellobiose produced several positive colonies. However, subculture of these colonies produced colonies of similar appearance but no longer PCR-positive for UBA3401, suggesting that the growth of this bacterium may require a syntrophic partner.

(a) Generation of UBA3401-specific primers targeting a unique region in the databank UBA3401 genome. (b) Gel-based detection of UBA3401 using primers generated in (a). Sample lanes were: (1) Lachnospiraceae-enriched consortia, (2) feces from germ-free mice used in limiting dilution transplants of UBA3401 that qPCR suggested may be mono-associated with UBA3401 at low abundance, and (3) ASF mouse administered fecal sample from lane 2. (c) Metagenomic sequencing analysis of the sample from lane 3. Results indicated that UBA3401 was the predominant non-ASF taxa in “UBA3401-enriched” ASF mice.

In parallel, we sought to isolate UBA3401 *in vivo* by limiting dilution fecal transplant into GF mice, followed by selecting mice with the highest relative abundance of UBA3401 based on Ct values using UBA-specific and universal 16S primers. Multiple rounds of this approach led to the generation of mice that were potentially mono associated with UBA3401 based on Ct values and that 16S sequencing yielded results consistent with mice being mono-associated with UBA3401 but the absolute amount of bacterial DNA in fecal samples from such mice was very low. These *in vivo* results further supported the suggestion that UBA3401 is highly recalcitrant to growing without the presence of other commensal members. However, the transfer of these feces harboring UBA3401 at low abundance to ASF mice led to an approximately 30-fold higher absolute abundance of UBA3401 and, concomitantly, a readily observable band on a gel. Sequence analysis indicated that such mice were indeed enriched in Lachnospiraceae, particularly UBA3401 (Figure 5(c)). Consequently, we used ASF mice as a means of propagating UBA3401.

### UBA3401 genome and potential mechanisms of colonization resistance

**Figure 6.**
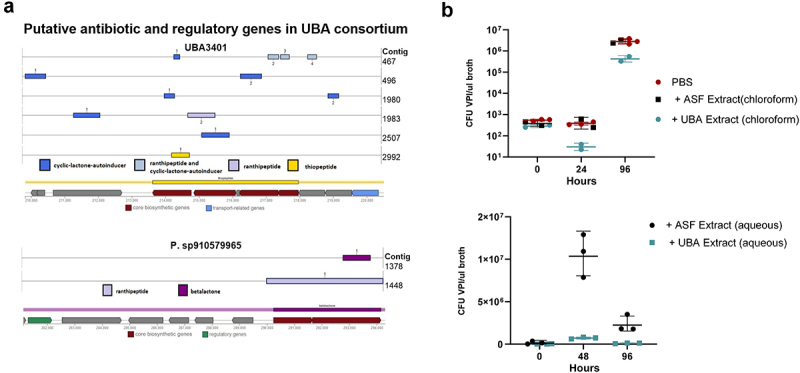
*In silica* and *in vitro* analysis indicate UBA3401 may directly impede *C. difficile* growth.

Feces from UBA3401-colonized ASF mice were further analyzed by in-depth shotgun and long-read metagenomic sequencing. Sequences were assembled to generate a UBA3401 genome estimated to approach 100% completion with 4.11% contamination. The genome appeared broadly similar to other Lachnospiraceae; however, analysis of the genome revealed the presence of a region closely homologous to a known thiopeptide bacteriocin gene cluster (Figure 6(a)). The putative thiopeptide cluster was organized in a manner that was consistent with known thiopeptide biosynthetic gene clusters (BGC) including a YcaO enzyme at the end of the BGC.^24^ We also noted an ABC transporter and efflux transporter located immediately downstream of the predicted thiopeptide BGC. Additionally, multiple genes implicated in the regulation of biosynthesis such as cyclic lactone autoinducers, along with quorum sensing ranthipeptide genes^25,26^ were found in the UBA3401 genome. Furthermore, a species that was also present in the UBA3401/ASF feces, namely *Paralachnospira sp910588405*, was found to have potential genes predicted to code for betalactone antibiotic production and ranthipeptide quorum sensing. These genomic elements suggest that UBA3401 might release products with direct anti *C. difficile* action. To test this notion, we mixed early log phase *C. difficile* cultures with extracts of feces from ASF and UBA3401-enriched ASF mice and monitored *C. difficile* growth. We found that, relative to ASF fecal extracts, the addition of fecal extracts from UBA3401/ASF mice reduced *C. difficile* growth *in vitro*, thus supporting the notion that UBA3401 may limit *C. difficile* growth via secreted products (Figure 6(b)). As an additional approach to glean insights into how UBA3401 might impede *C. difficile*, we functionally profiled our metagenomic data from Figures 3 and 4, seeking to identify functional changes as UBA3401 replaced *C. difficile*. We observed that increases in Lachnospiraceae UBA3401 correlated with an increase in unmapped genes (Figure S1), highlighting the difficulty of attaining better mechanistic understanding of how such colonization impacts microbiota function.

(a) Analysis of the UBA3401 genome identified putative antimicrobial genes as discussed in the text. (b) Extracts of ASF/UBA3401-colonized mice, but not ASF mice, impede *C. difficile* growth *in vitro*. Low-density cultures of *C. difficile* VPI10463 were administered extracts of feces from UBA and UBA3401 mice as described in Methods. *C. difficile* levels were measured by PCR at indicated time points. * Indicates a significant difference between ASF and ASF/UBA3401, *p* < 0.05.

### UBA3401 protects mice against C. difficile

**Figure 7.**
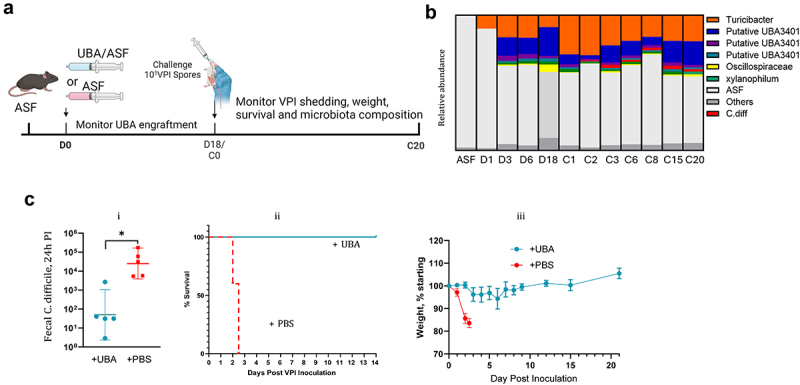
UBA3401 administration protects against CDI.

Lastly, we sought to examine the potential of UBA3401 to protect against *C. difficile* colonization and disease *in vivo*. Groups of ASF mice were administered cecal suspensions generated from ASF or UBA3401/ASF mice (Figure 7(a)). Monitoring UBA3401 engraftment by PCR found it was detectable at low levels within a few days post-administration. It then increased slowly over the course of 2 weeks, at which point it plateaued. Monitoring microbiota composition via 16S rRNA gene sequencing (used because of its lower cost compared to shotgun metagenomic sequencing), pre- and post-administration of the cecal transplants, supported the notion that UBA3401 was the predominant, albeit not the only bacteria introduced into the ASF microbiota (Figure 7(b)). In particular, we noted the appearance of *Turicibacter*, frequently observed in our various attempts to isolate UBA3401 at early time points preceding growth of UBA3401. 20 d post-microbiota transplant, mice were challenged with 10^5^ spores of *C. difficile*. As expected, recipients of ASF microbiotas remained highly prone to CDI with all mice displaying high levels of fecal *C. difficile* 1 d post-challenge. Concomitantly, such mice appeared overtly ill, lost weight, and died within 3 d (Figure 7(c)). In contrast, recipients of UBA3401-containing microbiotas displayed comparatively lower *C. difficile* loads and lacked clinical indices of disease. Yet, analysis of their feces by sequencing and PCR found that, unlike in the above-described experiments wherein ASF mice were administered whole Clostridia preps, recipients of UBA3401-enriched microbiotas did not completely clear the pathogen. Nonetheless, collectively, these results accord with UBA3401 providing colonization resistance against *C. difficile*.

(a) Experimental design schematic. (b) Microbiota analysis via 16S sequencing of feces from ASF mice on the indicated day following the administration of UBA3401-containing feces (d) and, subsequently, days following *C. difficile* administration (c). (c) Consequences of administering ASF3401 feces to ASF mice on *C. difficile* infection. (i) Fecal *C. difficile* level 24 h following inoculation with 10^5^ VPI10463 spores. (ii) Survival. (iii) Body weight. Data are means ± SD (*n* = 5, 3 M/2F). * Indicates statistical significance, *p* < 0.05.

## Discussion

*C. difficile* remains a major recalcitrant public health menace. Novel approaches to prevent and treat primary and recurrent infections are needed. We hypothesize that key insights into developing new strategies to combat *C. difficile* might come from a better understanding of how the gut microbiota provides colonization resistance against this pathogen. Attaining such understanding has been stymied by the complexity and heterogeneity of the gut microbiota. This study sought to surmount this hurdle via the use of gnotobiotic mice that were colonized with a minimal microbiota, specifically ASF mice, which, in contrast to germfree mice, are relatively immunologically normal. We found that ASF mice were highly prone to *C*CDI without the use of antibiotics, rapidly succumbing to high-dose *C. difficile* challenge and developing chronic *C. difficile* infections to lower *C. difficile* inoculums. The latter was associated with visually evident illness but, interestingly, not lastingly overt colitis for reasons that are unclear but may conceivably reflect a host mechanism to suppress the acute severe inflammation that might have been present at early times following colonization. Regardless, we found such chronically infected mice could serve as a platform to identify bacteria capable of preventing or clearing *C. difficile* infection. Specifically, we found that a complex undefined preparation of Clostridia provided stark protection against CDI and that a significant portion of this protection could be recapitulated by a highly fractionated derivative of this, namely one enriched in Lachnospiraceae UBA3401. We hypothesize that understanding how UBA3401 impedes CDI might suggest novel approaches to treat this condition. Furthermore, we envisage that pure UBA3401 cultures and/or spores might, if attainable, serve to treat and/or prevent CDI.

Some bacteria, for example, segmented filamentous bacteria, that are recalcitrant to growth *in vitro* achieve high levels when mono-colonizing mice, suggesting a requirement for a host-derived metabolite.^27^ In contrast, limiting dilution transplant of UBA3401-containing feces resulted in mice that were, based on qPCR, seemingly mono-associated with this microbe, but absolute levels of UBA3401 in such mice were extremely low suggesting its growth might require metabolites provided by other bacteria. That UBA3401 thrived in ASF mice accords with this notion. In this scenario, UBA3401-supporting bacteria may include both ASF species and/or other bacteria in the original consortium that engrafted with UBA3401 and may, themselves, be supported by ASF bacteria. Extensive efforts to culture UBA3401 were unsuccessful. Indeed, we designed a variety of media types seeking to promote the growth of UBA3401, including the use of gentamicin and aztreonam to suppress aerobic and gram-negative microbes respectively, blood and taurocholate to facilitate and induce the growth of endospores. On several occasions, colonies initially testing positive for UBA3401 were obtained but, following subculture, they had 16S sequences that indicated they were not UBA3401 but were consistent with *Blautia* species thus suggesting that UBA3401 might closely associate with *Blautia* species. On the other hand, the absence of UBA3401 in such sub-cultures indicates such *Blautia* were not sufficient to support UBA3401 growth. Supplementing growth media with extracts from ASF mice was also to no avail, suggesting that the assistance this bacterium attains from other microbes and/or the host may be short-lived and/or require contact or close proximity for delivery. The proliferation of *Turicibacter* prior to the growth of UBA3401 may reflect a syntrophic role in UBA3401 colonization: the genus *Turicibacter* is associated with extensive modification of host lipids and bile acids into novel products^28^ with bile acids or lipids modified by *Turicibacter* possibly being necessary for the growth of UBA3401.

**Figure 8.**
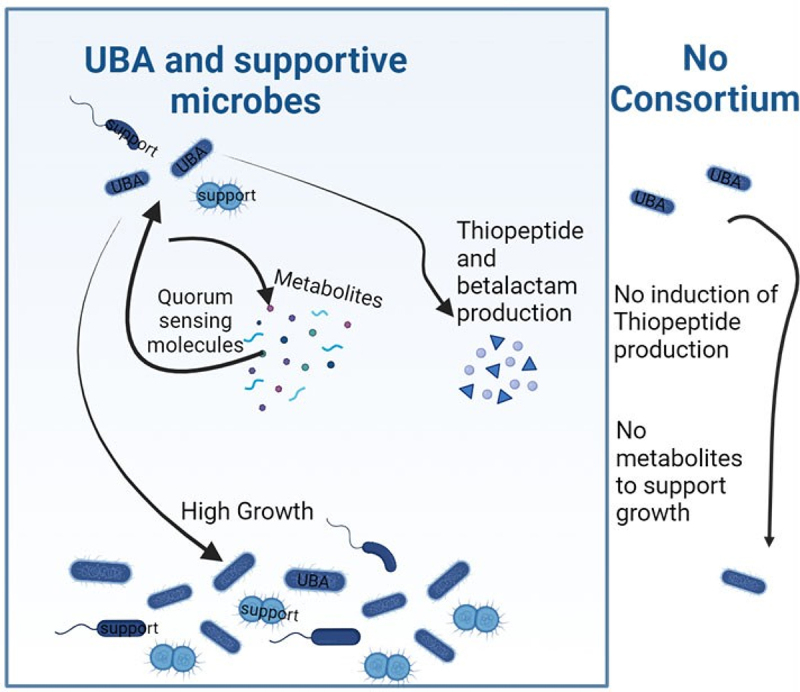
Hypothetical model of UBA3401 quorum-mediated protection against CDI.

How UBA3401 might limit *C. difficile* colonization, and consequently disease, is not yet clear, but analysis of the UBA3401 genome and our experimental observations suggest this bacterium may secrete factors that directly impede *C. difficile* growth. The UBA3401 genome contains an array of cyclic lactone autoinducers, which are associated with, and are thought to regulate bacteriocin production.^29,30^ Moreover, this segment of the UBA3401 genome encodes a secreted thiopeptide predicted to have direct antibacterial activity. Indeed, the thiopeptide spectrum of activity is directed against gram-positive microbes with derivatives known to have activity against *C. difficile*.^31^ In accordance with this notion, extracts of feces from UBA3401/ASF mice directly suppressed *C. difficile* growth *in vitro*. Cyclic lactone autoinducers also mediate quorum sensing-mediated gene expression and, consequently, may explain why some aspects of UBA3401’s activity are reminiscent of quorum-induced phenotypes.^32^ In particular, following the administration of UBA3401-enriched microbes into ASF mice, we’ve observed a considerable lag in UBA3401 growth and, moreover, in its protection against CDI. Hence, we envisage that, following its administration to mice, UBA3401 initially proliferates slowly until other, yet-to-be-defined bacteria that congregate with UBA3401, become established, followed by enhancement of UBA3401 growth and protection against *C. difficile*, analogous to other examples of bacterial quorum sensing amensalism where production of bacteriocin is only induced after signaling mass within a microbial community is reached (schematized in Figure 8).^33,34^ Indeed, induction of bacteriocin production by mixed species consortia is widespread in nature with the addition of a supportive species greatly increasing bacteriocin production.^35,36^

Discussed in the text.

That both UBA3401 growth and expression of bacteriocins appear to be dependent upon other microbes underscores the complexity of the gut microbiota and, consequently, the difficulty in seeking to precisely manipulate it. Indeed, hosts with normal microbiotas that have not been perturbed by antibiotics show near-complete resistance to *C. difficile* colonization. Furthermore, while undefined fecal transplants are highly effective at preventing *C. difficile* recurrence, replicating such efficacy with fully defined bacterial cocktails has proven far more difficult. In this context, we conclude that UBA3401 may be one bacterium capable of strongly impeding *C. difficile*. Consequently, better understanding the growth of this bacterium, regulation of its gene expression, and the mechanism by which it impedes *C. difficile* growth may eventuate in better and safer strategies to counteract this pathogen.

## Supplementary Material

Supplemental Material

## Data Availability

Sequencing data were deposited to the European Nucleotide Archive under accession PRJEB73455 (16S and Illumina HiSeq metagenomic reads) and accession PRJEB73515 (NextSeq and PacBio data for hybrid MAG assembly). All other data utilized are within the manuscript itself.
