# Supplementary material for: Prevention and cure of murine C. difficile infection by a Lachnospiraceae strain
Source: Gut Microbes. 2024 Aug 27;16(1):2392872. doi: 10.1080/19490976.2024.2392872 (PMC11352710; doi:10.1080/19490976.2024.2392872)
Supplement: Supplemental Material [file KGMI_A_2392872_SM6507.zip › Supplementary files-1.docx]

**SUPPLEMENTAL FIGURE LEGENDS**

**Figure S1:** Functional profiling of metagenomic sequencing data from Figure 3 via HUMAnN classified UniRef50 clusters. Data are shown as relative percent of unmapped categories with no corresponding UniRef50 cluster, the top 200 most abundant UniRef clusters (as cycling colors), and all other rarer clusters grouped as All Other Clusters. The categories of Clostridia Prep, Heat-treated (HT) Prep, Culturable Prep, and PBS are as described in Figure 3.

**Figure S2:** Functional profiling of metagenomic sequencing data from Figure4 via HUMAnN classified UniRef50 clusters. **A.** Data are shown as relative percent of unmapped categories with no corresponding UniRef50 cluster, the top 200 most abundant UniRef clusters (as cycling colors), and all other rarer clusters grouped as All Other Clusters. The categories of Lachnospiraceae Consortium and PBS are the same as described in Figure 4. **B.** Jitter plot showing % reads of *Clostridium* phage phiCD6356 protein, which was, following Holm correction for multiple comparison testing, the only Uniref50 cluster that was statistically significantly different between the Lachnospiraceae Consortium and PBS control groups.

Figure S1


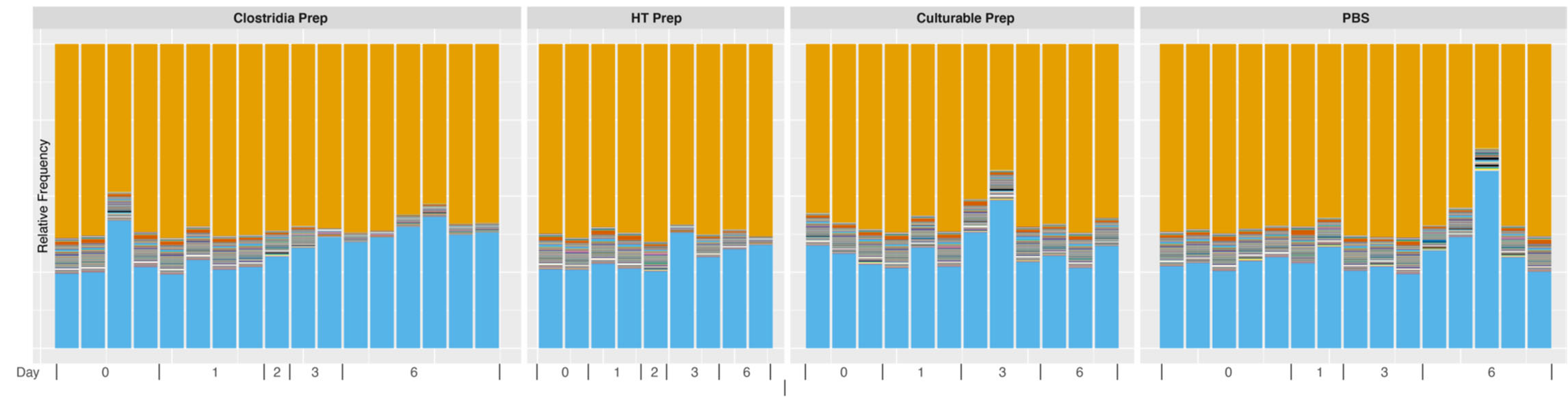


Figure S2


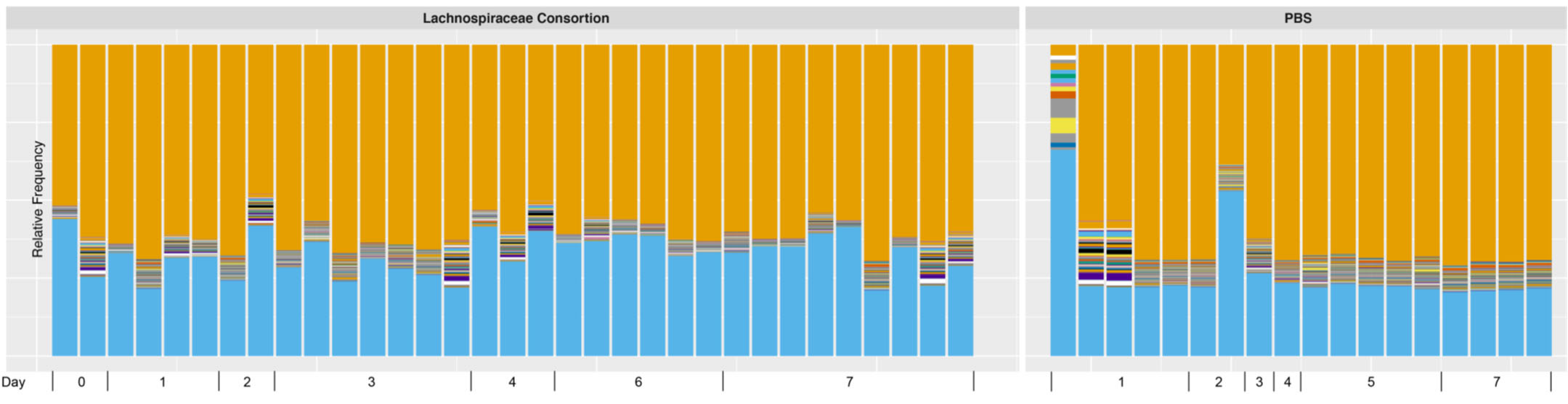
**A**


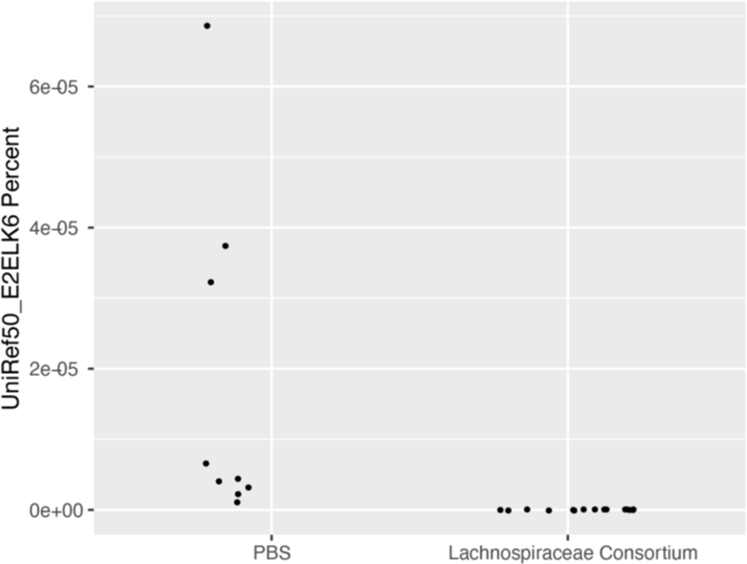
**B**
